# Multiple *de novo* copy number variations in two subjects with developmental problems and multiple congenital anomalies

**DOI:** 10.1186/1753-6561-6-S6-P25

**Published:** 2012-10-01

**Authors:** Pengfei Liu, Klaudia Walter, Karin Writzl, Violet Gelowani, Sarah Lindsay, Claudia MB Carvalho, Marjorie Withers, Joanna Wiszniewska, Ankita Patel, Bernd Rautenstrauss, Matthew E Hurles, James R Lupski

**Affiliations:** 1 Department of Molecular and Human Genetics, Baylor College of Medicine, Houston, TX, USA; 2 Department of Pediatrics, Baylor College of Medicine, Houston, TX, USA; 3 Texas Children's Hospital, Houston, TX, USA; 4 Wellcome Trust Sanger Institute, Hinxton, UK; 5 Institute of Medical Genetics, UMC, Ljubljana, Slovenia; 6 Medical Genetics Center, Bayerstrasse 3-5, Munich, Germany

## Background

*De novo* copy number variation (CNV) can occur constitutionally in gametogenesis or in early development leading to sporadic genomic disorders. Such *de novo* CNVs appear to also be important in somatic mutagenesis relevant to cancer and population events important to species evolution. Since large pathological CNVs are rarely observed at more than one locus in a single patient, and are often *de novo*, current efforts in understanding their molecular features and underlying mechanisms have relied on comparing CNVs from different individuals. Therefore, knowledge regarding size, mechanism and spatial distribution *of de novo* genomic rearrangements in a single genetic background is lacking.

## Materials and methods

Two subjects with developmental problems and multiple congenital anomalies were identified by clinical array comparative genomic hybridization (aCGH) to have more than five *de novo* CNVs larger than 500 kb. Custom-designed aCGH and whole genome SNP arrays were used to fine map large *de novo* CNVs in both of the subjects and determine parental origins, whereas breakpoint sequencing was performed to glean insights into mechanism. In order to characterize smaller sized CNVs not efficiently interrogated by aCGH, Illumina whole genome sequencing was performed on both subjects to obtain a sequence coverage of more than 30×.

## Results

We report two subjects with a constitutional 'CNV mutator' phenotype, in whom multiple *de novo* rearrangements were observed on different chromosomes. Such observations are distinguishable from the phenomenon of chromothripsis in which the multiple CNV changes concentrate on one chromosome [1]. Subject no. 1 carried 8 large (>100 kb) copy number gains, ranging from 104 kb to 6.4 Mb. Subject no. 2 carried 11 large copy number gains, ranging from 211 kb to 4.7 Mb. Breakpoint sequencing analysis showed that microhomologies and breakpoint complexities are the prevailing features left at rearrangement traces, suggesting that the rearrangements were likely produced by replication mechanisms such as fork stalling and template switching and/or microhomology-mediated break-induced replication (FoSTeS/MMBIR). Haplotype analysis in subject no. 1 revealed that the duplicated or triplicated materials were derived from both the paternal chromosome and the maternal chromosome, suggesting a postzygotic timing of the mutations.

## Conclusions

Our results document a genome-wide spectrum of *de novo* CNVs in a 'CNV mutator' phenotype background, and we suggest that errors in the cellular DNA replication machinery could lead to multiple independent *de novo* rearrangements. Our findings have important implications for genomic disorders, cancer and evolution.

## References

[B1] Liu P Carvalho CM Hastings P Lupski JR Mechanisms for recurrent and complex human genomic rearrangements Curr Opin Genet Dev 2012 22 211 220 10.1016/j.gde.2012.02.012 22440479 PMC3378805

